# Effects of Erythropoietin Payment Policy on Cardiovascular Outcomes of Peritoneal Dialysis Patients: Observational Study

**DOI:** 10.2196/18716

**Published:** 2020-12-17

**Authors:** Ying-Hui Hou, Feng-Jung Yang, I-Chun Lai, Shih-Pi Lin, Thomas TH Wan, Ray-E Chang

**Affiliations:** 1 Department of Health Industry Management School of Healthcare Management Kainan University Taoyuan Taiwan; 2 Renal Division Department of Internal Medicine National Taiwan University Hospital Yun Lin Branch Douliu Taiwan; 3 School of Medicine, College of Medicine National Taiwan University Taipei Taiwan; 4 Center for Drug Evaluation Taipei Taiwan; 5 Institute of Health Policy and Management College of Public Health National Taiwan University Taipei Taiwan; 6 Public Affairs PhD Program College of Health and Public Affairs University of Central Florida Orlando, FL United States

**Keywords:** erythropoietin, cardiovascular disease, peritoneal dialysis, diabetes mellitus

## Abstract

**Background:**

The change in the reimbursement policy of erythropoietin administration to patients receiving peritoneal dialysis by the Taiwan National Health Insurance (NHI) system provided a natural experimental venue to examine whether cardiovascular risk differs when maintaining the hematocrit (Hct) level below or above 30%.

**Objective:**

The aim of this study was to analyze the impact of loosening the erythropoietin payment criteria for peritoneal dialysis patients on their cardiovascular outcomes.

**Methods:**

Two cohorts of incident peritoneal dialysis patients were identified according to the time before and after relaxation of the NHI’s erythropoietin payment criteria, designated cohort 1 (n=1759) and cohort 2 (n=2981), respectively. The cohorts were matched according to propensity scores (1754 patients in each cohort) and then followed up for cardiovascular events, which were analyzed with Cox regressions.

**Results:**

For the composite cardiovascular endpoint, patients in cohort 2 had a significantly lower risk than those in cohort 1. However, subgroup analysis showed that this risk reduction was observed only in patients with diabetes.

**Conclusions:**

After loosening erythropoietin payment criteria, reduced cardiovascular risks were observed, particularly for patients with diabetes. These results indicate that it is crucial to maintain an Hct level above 30% to reduce the cardiovascular risk in patients with diabetes undergoing peritoneal dialysis.

## Introduction

Erythropoietin is a major regulatory hormone of erythrocyte production that is produced from the kidney, and its levels are decreased in patients with chronic kidney disease (CKD). A reduction in erythropoietin further decreases erythrocyte survival and leads to a chronic inflammatory status that contribute to anemia. Administration of exogenous erythropoietin for CKD patients, especially those receiving dialysis, is the standard treatment for anemia.

Early studies showed that the use of erythropoietin tended to increase the hematocrit (Hct) target to the normal level (ie, 40.5% for men and 36% for women). However, more recent large, randomized outcome trials [1-3] showed that elevating the Hct level above 36% compared to maintaining Hct in the range of 30%-36% was associated with a higher risk of cardiovascular events for patients with CKD. These findings led to establishing the limitation of the Hct upper bound; however, the optimal Hct target remains debatable. The recommendations from the National Kidney Foundation-Kidney Disease Outcomes and Quality Initiative [4] and Taiwan’s nephrology professionals [5] suggest maintaining the level of Hct between 33% and 36%.

The public statement of the European Medical Agency in 2007 concluded that the target Hct range should be 30%-36% [6]. The 2011 safety announcement of the US Food and Drug Administration recommended reducing or interrupting erythropoietin administration if the Hct level approaches or exceeds 33% for patients undergoing dialysis [7]. The recommendation from the Kidney Disease Improving Global Outcome in 2012 Clinical Practice Guideline was to maintain Hct below 34.5% [8]. Accordingly, an Hct range of 30%-36% might be considered the minimal bandwidth to accommodate all of these recommendations.

To reduce the cost of providing end-stage renal disease (ESRD) treatments while maintaining, or preferably improving, patient care, the US Center for Medicare and Medicaid (CMS) implemented the ESRD Prospective Payment System, known as the “expanded ESRD bundle,” on January 1, 2011 [9]. Moreover, in response to a quality incentive program (QIP) required by US congress, two quality measures of anemia management were established to identify poor performance: patients with a hemoglobin (Hb) level less than 10 g/dL and those with an Hb level greater than 12 g/dL [9]. These Hb levels are equivalent to an Hct level less than 30% and above 36%, respectively, since 1 g/dL of Hb is equal to 3% Hct. However, the CMS retired the measure of an Hb level less than 10 g/dL in its later QIP requirements [10,11]; that is, dialysis facilities would receive no penalties for patients with Hb levels lower than 10g/dL, who might be spotted more often in the future. The elimination of penalties for the lower bound of Hb levels has indeed removed the financial incentives to provide costly erythropoietin treatment, while raising some concerns about patient care [12]. Nevertheless, it remains unclear whether patients with an Hb level lower than 10 g/dL or an Hct level lower than 30% have a higher risk of adverse events, which is a logical inquiry that warrants further investigation.

Limited studies have reported cardiovascular events or mortality associated with Hct levels lower than 30%. Studies comparing dialysis patients with an Hct level maintained below 30% to those with Hct levels maintained in the range of 30%-36% showed no significant difference in adverse outcomes [13-15]. However, more recent studies [1-3] comparing the risk of pushing Hct levels above 36% with those maintained between 30%-36% included a larger sample size of more than 1200 patients with a follow-up period of more than 14 months, in contrast to the early studies with a relatively small sample size of 152 patients or less and a short follow-up period of 6-9 months. Moreover, the design of these studies was not specifically focused on assessing this question. Recently, the change in the reimbursement policy of erythropoietin administration to patients undergoing peritoneal dialysis by the Taiwan National Health Insurance (NHI) system provides a natural experimental venue for directly examining this clinical research issue.

The incidence and prevalence rates of ESRD in Taiwan have been ranked at the top internationally since 2001 [16], placing an immense burden of caring and funding for ESRD patients on the Taiwan NHI system. The low renal transplant rate, at less than 1% annually [17], results in nearly all of Taiwan’s ESRD patients relying on dialysis treatments to prolong their lives, with more than 93.5% of ESRD patients receiving hemodialysis treatments in 2004 [18]. To increase peritoneal dialysis utilization, Taiwan’s NHI has introduced a series of encouragement policies since 2005, including loosening the reimbursement criteria. Before November 1, 2006, the treatment of erythropoietin to a patient undergoing peritoneal dialysis could only be reimbursed by the NHI if the patient’s Hct level was ≤30% and they were receiving a maximal monthly erythropoietin dosage of 20,000 U epoetin alfa/beta or 100 μg darbepoetin alfa. After November 1, 2006, the Hct level at which erythropoietin administration could be reimbursed was relaxed to ≤36% with the same maximal monthly erythropoietin dosage requirements. Subsequent to this relaxation of erythropoietin administration criteria, the Hct levels for both prevalent and incident peritoneal dialysis patients increased from 28%-29% to 30%-31% [19-21].

The main purpose of this study was to analyze the impact of loosening the erythropoietin administration criteria for patients undergoing peritoneal dialysis in Taiwan with a focus on exploring the risk of cardiovascular events when maintaining Hct at 30%-31% as compared to 28%-29%.

## Methods

### Ethics Statement

Data were obtained from the National Health Insurance Research Database [22], which are accessible to researchers after ethical and scientific review processes. Prior to applying for this access, this study was approved by the ethical review board of National Taiwan University Hospital (NTUH-REC No. 201406018W). There are 27 institutional review boards capable of issuing approvals, and all are supervised and regulated by the Taiwan Ministry of Health and Welfare. To protect individuals’ confidentiality, all datasets in the Data Science Centre are pseudonymized. Personal ID, birth date, and names are encrypted, and this deidentification process was approved by an independent third party. We performed data analysis in the branches of the Data Science Centre. The analyzed results were also examined by the Data Science Centre before exporting. The Institutional Review Board verified the anonymity of data analysis performed in this study. All research procedures followed the directives of the Declaration of Helsinki.

### Study Design

This was an observational study designed to compare the cardiovascular events of two cohorts of newly treated (incident) patients undergoing peritoneal dialysis before and after relaxation of the NHI’s erythropoietin payment criteria. Cohort 1 included dialysis patients who started to receive maintenance peritoneal dialysis treatments during a specified period of 28 months before relaxation of the NHI’s erythropoietin payment criteria. To ensure an adequate observation period, this cohort was followed up for an additional 14 months after the month in which the last patient was enrolled in the study. Cohort 2 included incident dialysis patients who started to receive maintenance peritoneal dialysis treatments within a 28-month time interval after relaxation of the NHI’s erythropoietin payment criteria. Additional 14-month follow-up observations were also made after the month in which the last patient of this cohort was enrolled in the study. We set a 6-month time lag between the initiation of relaxing the erythropoietin payment criteria and the time that the first patient was enrolled in cohort 2 to accommodate possible adaptations of the physician prescribing practices to the new policy.

Because of potential imbalances in the distributions of many measured and unmeasured baseline covariates between the two cohorts, propensity score (PS) analysis, which was developed by Rosenbaum et al [23], was used in this study. Thus, the influence of any potential enrollment biases between these two cohorts was attenuated through a PS-matching approach and identification of patients with comparable characteristics in the two cohorts. This study defined PS as the probability of a patient having experienced a cardiovascular event. Patients in cohorts 1 and 2 were matched with PS scores estimated by age, sex, and the comorbidity index with the Greedy nearest neighbor algorithm [24]. The comorbidity index was developed by Liu et al [25] specifically for the US Medicare dialysis population and was subsequently validated for Taiwanese dialysis patients [26].

After matching with the PS, patients were followed up until experiencing either one of the following three events: (1) the occurrence of cardiovascular endpoints, (2) change to hemodialysis, or (3) the data cutoff point (October 31, 2006 for cohort 1 and October 31, 2010 for cohort 2), whichever occurred earlier. Survival analysis models were then employed to investigate the differences in the risk of cardiovascular events between the two cohorts of incident peritoneal dialysis patients. Baseline demographics and comorbid conditions were used as covariates in the statistical analyses. Monthly erythropoietin doses administered to patients of cohort 1 and cohort 2 during the follow-up period were compared to examine a difference between the two cohorts of incident peritoneal dialysis patients. In calculation of erythropoietin dosage, epoetin alfa and epoetin beta were considered to be equivalent, whereas darbepoetin alfa was converted to epoetin alfa based on the equivalence of 1 μg of darbepoetin alfa to 200 U of epoetin alfa [27].

Cardiovascular risk could be affected by treatments with concomitant medications related to cardiovascular comorbidities. Therefore, patients taking medications related to cardiovascular comorbidities during the follow-up period in the two cohorts were also examined. The concomitant medications related to cardiovascular comorbidities were identified by corresponding Anatomical Therapeutic Chemical classification codes, including acetylsalicylic acid (B01AC06) or clopidogrel (B01AC04), angiotensin-converting enzyme inhibitors (C09A) or angiotensin receptor blockers (C09C), beta blockers (C07), calcium channel blockers (C08), and statins (C10AA). A patient who received such medication for any of the 3 months during the follow-up period would be considered to be under treatment of concomitant medications related to cardiovascular comorbidities.

Finally, in addition to administering erythropoietin, because the patient’s Hct level could also be affected by the use of iron and red cell transfusion, the differences in iron and red cell transfusion were compared between patients in the two cohorts.

### Patient Selection

Incident peritoneal dialysis patients were identified from the claim data of entire beneficiaries covered by the NHI system from 2003 to 2010. Collection and analysis of the NHI claimed data were approved by the National Taiwan University Hospital Human Research Ethics Committee. The analyses were performed on deidentified data extracted from the NHI research database compiled by Taiwan National Health Research Institutes. A patient receiving over 90-day consecutive dialysis treatments and with peritoneal dialysis performed on day 90 and thereafter was considered to be an incident peritoneal dialysis patient in this study. Cohort 1 included patients who received dialysis as of the 90th day between May 1, 2003 and August 31, 2005, and cohort 2 included patients who received dialysis as of the 90th day between May 1, 2007 and August 31, 2009. Young patients (under 20 years) were excluded because comorbidities differed between pediatric and adult patients. There were 1759 patients in cohort 1 and 2981 patients in cohort 2. After PS-based matching, each cohort contained 1754 patients.

### Statistical Analyses

The primary outcome measure was a composite cardiovascular endpoint, defined as myocardial infarction, heart failure hospitalization, stroke, or death. Myocardial infarction was defined by International Classification of Diseases, Ninth Revision (ICD-9) codes 410 and 411 in the hospital discharge diagnosis. Heart failure hospitalization was defined by ICD-9 hospital discharge diagnosis codes 398.91, 422, 425, 428, 402.x1, 404.x1, 404.x3, and V42.1. Stroke was defined by ICD-9 hospital discharge diagnosis codes 433, 434, 436, 437.0, and 437.1. For the primary outcome measure, all patients in both cohorts were followed up until the occurrence of myocardial infarction, heart failure hospitalization, stroke, or death, whichever occurred earlier. Secondary outcomes were the individual components of the composite primary outcome: myocardial infarction, heart failure hospitalization, stroke, and death. Each patient was followed up until the occurrence of each cardiovascular event. Data on patients who did not have an event were censored at the data cut-off point or date of transition to hemodialysis, whichever occurred earlier.

The selection and analyses of primary and secondary endpoints of cardiovascular risk in this study were the same as those adopted in previous large-scale studies [1-3]. In addition to cardiovascular events, death was also considered an important clinical endpoint in the evaluation of cardiovascular risk because reducing mortality is an ultimate goal of reducing cardiovascular risk. Using a composite primary endpoint with each component evaluated as the secondary endpoint analysis is commonly adopted by many clinicians [2,3], such as in pivotal studies of new drug applications. This allows for a thorough evaluation of the contribution of each component of the composite primary endpoint and avoids any biases introduced by a dominating component.

The Cox proportional hazards model was employed to estimate the cardiovascular risk between the two cohorts. Estimated hazard ratios (HRs) for cohort 2 relative to cohort 1 and 95% CIs were calculated. To obtain more insightful results, patients were further stratified by diabetes status; Cox regression analyses for patients with and without diabetes were performed separately. All analyses were performed using SAS software, version 9.1.

## Results

### Patient Selection

Table 1 shows the baseline demographics and comorbid conditions of the equal number (n=1754) of incident peritoneal dialysis patients in the two cohorts. No statistically significant differences were observed, suggesting that patients in the two cohorts appeared to be similar in terms of age, gender, and comorbid conditions at baseline. There were also no significant differences in the usage of any concomitant medication related to cardiovascular comorbidities between the two cohorts.

**Table 1:** Baseline demographics and concomitant medications during the follow-up period in cohort 1 and cohort 2 after matching with the propensity score.

Characteristic		Matched^a^ cohort 1 (n=1754)	Matched cohort 2 (n=1754)	*P* value^b^
Female, n (%)		994 (56.67)	991 (56.50)	.84
Age (years), mean (SD)		52.96 (15.36)	52.87 (15.02)	.33
**Age group (years), n (%)**				
	20-39	326 (18.59)	327 (18.64)	
	40-49	390 (22.23)	384 (21.89)	
	50-59	431 (24.57)	444 (25.31)	
	60-69	320 (18.24)	324 (18.47)	
	≥70	287 (16.36)	275 (15.68)	
Comorbidity index, mean (SD)		2.52 (1.72)	2.52 (1.79)	.80
**Comorbidity index, n (%)**				
	0	401 (22.86)	401 (22.86)	
	1	268 (15.28)	269 (15.34)	
	2	324 (18.47)	323 (18.42)	
	3	245 (13.97)	243 (13.85)	
	4	180 (10.26)	182 (10.38)	
	5	148 (8.44)	148 (8.44)	
	6	94 (5.36)	94 (5.36)	
	7	49 (2.79)	50 (2.85)	
	8	24 (1.37)	23 (1.31)	
	9	10 (0.57)	10 (0.57)	
	≥10	11 (0.63)	11 (0.63)	
**Baseline comorbidity, n (%)**				
	Atherosclerotic heart disease	327 (18.64)	320 (18.24)	.49
	Congestive heart failure	192 (10.95)	192 (10.95)	>.99
	Cerebrovascular accident/transient ischemic attack	273 (15.56)	268 (15.28)	.67
	Peripheral vascular disease	250 (14.25)	253 (14.42)	.76
	Other cardiac disease	220 (12.54)	223 (12.71)	.75
	Chronic obstructive pulmonary disease	106 (6.04)	110 (6.27)	.59
	Gastrointestinal bleeding	212 (12.09)	207 (11.80)	.65
	Liver disease	200 (11.40)	204 (11.63)	.66
	Dysthymia	60 (3.42)	56 (3.19)	.48
	Cancer	149 (8.49)	151 (8.61)	.80
	Diabetes	581 (33.12)	584 (33.30)	.82
	Hypertension	1297 (73.95)	1305 (74.40)	.70
	Atrial fibrillation	19 (1.08)	15 (0.86)	.33
	Coronary artery bypass graft	134 (7.64)	128 (7.30)	.59
	Myocardial infarction	22 (1.25)	21 (1.20)	.89
**Concomitant medications, n (%)**				
	Acetylsalicylic acid or clopidogrel	1369 (78.05)	1355 (77.3)	.39
	ACEIs^c^ or ARBs^d^	637 (36.32)	631 (35.97)	.38
	Beta blockers	589 (33.58)	586 (33.41)	.48
	CCB^e^	683 (38.94)	693 (39.51)	.37
	Statins	509 (29.02)	504 (28.73)	.32
Oral iron usage, n (%)		72 (4.10)	69 (3.93)	.63
Intravenous iron usage, n (%)		794 (45.27)	772 (42.01)	.61
Red cell transfusions, n (%)		194 (11.06)	170 (9.69)	.09
Red cell transfusion units per patient per month, mean (SD)		0.059 (0.216)	0.044 (0.172)	.03
Oral iron dose per patient per month (mg), mean (SD)		25.06 (129.66)	23.39 (125.0)	.23
Intravenous iron dose per patient per month (mg), mean (SD)		106.54 (92.29)	98.91 (89.38)	.19
Erythropoietin^f^ usage per patient per month (U), median (IQR)		10,588^c^ (7750-13,280)^d^	12,379^c^ (8580-14,570)	<.001

^a^Matching with propensity score was based on age, sex, and comorbidity index using the Greedy method.

^b^Means (SD) were compared with the *t* test, n (%) values were compared with the proportion *z* test, and medians (IQR) were compared with the Wilcoxon rank-sum test.

^c^ACEIs: angiotensin converting enzyme inhibitors.

^d^ARBs: angiotensin receptor blockers.

^e^CCB: calcium channel blocker.

^f^Including epoetin alfa, epoetin beta, and darbepoetin alfa; epoetin alfa and beta were considered equivalent, and 100 μg darbepoetin was considered equivalent to 20,000 U erythropoietin according to the reimbursement criteria of the Taiwan National Health Institute.

### Erythropoietin Dosage

The median monthly erythropoietin dosage was significantly higher in cohort 2 than in cohort 1 (12,739 U vs 10,588 U, *P*<.001). The usage of iron supplements (both oral and intravenous) and red cell transfusions were comparable in the two cohorts (Table 1).

### Endpoint Evaluation

For the composite cardiovascular endpoint, the risk in cohort 2 was significantly lower after adjusting for age, sex, comorbidity index, diabetes mellitus, hypertension, history of coronary artery bypass graft, and congestive heart failure (Table 2). For each cardiovascular endpoint, the risk reduction in cohort 2 did not reach statistical significance.

**Table 2:** Comparison of primary and secondary endpoints between the cohorts.

Endpoint		Matched cohort 1 (n=1754), n (%)	Matched cohort 2 (n=1754), n (%)	Hazard ratio^a^ (95% CI)	*P* value
Primary endpoint: cardiovascular composite events		299 (17.05)	261 (14.88)	0.82 (0.69-0.98)	.04
**Secondary endpoints**					
	Myocardial infarction	40 (2.28)	36 (2.05)	0.81 (0.48-1.19)	.20
	Stroke	58 (3.31)	45 (2.57)	0.72 (0.50-1.12)	.15
	Heart failure hospitalization	173 (9.86)	162 (9.24)	0.76 (0.65-1.09)	.17
	Death	91 (5.19)	89 (5.07)	0.92 (0.68-1.24)	.59

^a^Adjusted for age, sex, comorbidity index, diabetes, hypertension, history of coronary artery bypass graft, and congestive heart failure.

In the subgroup analysis (Table 3), for patients that did not have diabetes, no significant difference in either the composite cardiovascular endpoint or any individual cardiovascular endpoint was observed between the two cohorts. However, for patients with diabetes, the risk of the composite cardiovascular endpoint was significantly lower in cohort 2. In addition, the risks of stroke and heart failure hospitalization were significantly lower in cohort 2 than those of cohort 1.

**Table 3:** Subgroup analysis according to diabetes status in comparing the endpoints between matched cohort 1 and cohort 2.^a^

Endpoint		Patients with diabetes^b^			Patients without diabetes^c^			
		Hazard ratio^d^ (95% CI)		*P* value		Hazard ratio^d^ (95% CI)		*P* value
Primary endpoint: Cardiovascular composite		0.74 (0.60-0.93)		.006		0.97 (0.74-1.27)		.82
**Secondary endpoints**								
	Myocardial infarction	0.67 (0.36-1.15)		.19		0.86 (0.33-2.25)		.76
	Stroke	0.61 (0.39-0.98)		.04		1.02 (0.51-2.04)		.93
	Heart failure hospitalization	0.72 (0.54-0.99)		.04		1.06 (0.74-1.51)		.76
	Death	1.07 (0.73-1.58)		.73		0.79 (0.49-1.26)		.27

^a^Patients in cohorts 1 and 2 were matched with the propensity score by age, sex, and comorbidity index using the Greedy method.

^b^Cohort 1, n=581; cohort 2, n=584.

^c^Cohort 1, n=1173; cohort 2, n=1170.

^d^Adjusted by age, sex, comorbidity index, hypertension, history of coronary artery bypass graft, and congestive heart failure.

## Discussion

### Summary

No statistically significant difference was observed for baseline comorbidities and concomitant medications in the follow-up period between the matched cohort 1 and cohort 2 (Table 1). This suggests that both cohorts had similar cardiovascular risk factors. After loosening erythropoietin payment criteria, the erythropoietin dosage increased and the cardiovascular risk decreased; however, the reduction in cardiovascular risk was observed only in patients with diabetes. In addition, among patients with diabetes, significant risk reduction was found not only for the composite cardiovascular endpoint but also for the individual secondary endpoints, including stroke and heart failure hospitalization. Since similar percentages of patients in matched cohort 1 and cohort 2 received oral and intravenous iron, and the oral and intravenous iron dosage was comparable between these two cohorts, it is reasonable to assume that the higher Hct level in matched cohort 2 might have resulted from the higher erythropoietin dosage. Similarly, the reduction in cardiovascular risk in matched cohort 2 may be related to the higher erythropoietin dosage and maintenance of an adequate Hct range.

### Comparison With Prior Work

Although previous findings that pushing Hct to more than 36% compared to 30%-36% tends to increase cardiovascular risk [1-3,7] have been widely accepted and recommended, there is a lack of sufficient evidence to demonstrate a difference in cardiovascular risk by maintaining Hct levels below 30% relative to 30%-36%. A few studies with small sample sizes and short follow-up periods showed no significant difference in cardiovascular risk or mortality for patients maintaining Hct below 30% compared to those maintaining Hct at 30%-36% [13-15]. Thus, these limitations have prevented investigators from detecting the potential difference in cardiovascular risk. By contrast, our national study showed that a lower cardiovascular risk is associated with increasing Hct from 28%-29% to 30%-31% for incident peritoneal dialysis patients in Taiwan. The number of subjects in our study was 3508 and the median follow-up duration was 23 months, which are comparable to those of more recent large-scale studies [1-3] with a sample size between 1265 and 4038 and median follow-up duration between 14 and 29 months.

### Principal Findings

Although the Hct data reported in the NHI beneficiaries claim database did not directly link to observations of patients’ Hct levels of this study, we used the data from the whole NHI population (census) and government documents publishing Hct statistics for dialysis patients supported by the NHI [19-21]. Moreover, from the governmental published data, the Hct levels of both prevalent and incident peritoneal dialysis patients were very similar (28.9% to 30.4% vs 29.1% to 30.4% from 2005 to 2008) and the Hct of both peritoneal dialysis patients with and without diabetes mellitus were also very similar (28.5% to 30.6% vs 28.3% to 30.3% from 2003 to 2008). Therefore, we assumed that the Hct levels of incident peritoneal dialysis patients in our study were similar to those reported in the government documents. After loosening the erythropoietin payment criteria, the Hct level of both prevalent and incident peritoneal dialysis patients increased from 28%-29% to 30%-31% [19-21].

In this study, the median erythropoietin dosage in cohort 2 (12,739 U) was significantly higher than that in cohort 1 (10,588 U); that is, there was a more than 20% increase in the dosage after loosening the erythropoietin reimbursement criteria. Given that the usage rates of iron supplements (both oral and intravenous) and red cell transfusions were comparable in the two cohorts, increased erythropoietin usage supports the assumption that the Hct of incident peritoneal dialysis patients also increased after loosening the erythropoietin payment criteria.

Because the reduction in cardiovascular risk was observed only in patients with diabetes, the difference in cardiovascular event risk reduction between patients with and without diabetes might not be the result of the Hct difference; indeed, the Hct was similar between peritoneal dialysis patients with (28.5%-30.6%) and without (28.3%-30.3%) diabetes from 2003 to 2008 [21]. Therefore, rather than analyzing the two subgroups (with and without diabetes) separately through a Cox proportional hazards model, we reanalyzed the nonstratified data through a Cox proportional hazards model with the addition of two more variables: one dichotomous variable for differentiating patients according to diabetes status and another interaction term between diabetes status and cohort. The estimate of diabetes status represented the cardiovascular risk of patients with diabetes relative to that of patients without diabetes in the time period of cohort 1, and the estimate of the interaction term measured the change in cardiovascular risk of patients with diabetes relative to that of patients without diabetes in the time period of cohort 2 compared to the time period of cohort 1. These results showed that the incident peritoneal dialysis patients with diabetes had a significant 78% higher cardiovascular risk than those of patients without diabetes. Although there was no significant difference in cardiovascular risk observed for our peritoneal dialysis patients without diabetes in cohort 2 (HR 0.974, 95% CI 0.84-1.05), the cardiovascular risk of the patients with diabetes in cohort 2 was significantly reduced by 22% (HR 0.78, 95% CI 0.61-0.94). This means that the cardiovascular risk of incident peritoneal dialysis patients with diabetes mellitus was 39% (1.78×0.78=1.39) higher than that of patients without diabetes in the time period of cohort 2, and was reduced by 78% in the time period of cohort 1. There was no significant difference in the erythropoietin dosages used for patients in the two cohorts according to diabetes status in either cohort (diabetes vs no diabetes median 10,726 U vs 10,525 U, *P*=.09 in cohort 1; 12,254 U vs 12,310 U, *P*=.17 in cohort 2). Given these findings and the similar Hct levels between the patients with and without diabetes, the observed increases in erythropoietin dosage and the Hct levels from below 30% to above 30% might benefit peritoneal dialysis patients with diabetes in terms of reducing the cardiovascular risk but would have no impact on the cardiovascular risk of patients without diabetes.

This finding has an important implication for policymakers for making decisions as to how to allocate health care resources and improve patient care in a cost-efficient manner, which is a major challenge for policymakers worldwide, including Taiwan and the United States. Based on these findings, Taiwan’s NHI policymakers should reconsider the relaxation of NHI’s reimbursement criteria to target only peritoneal dialysis patients with diabetes rather than applying these criteria universally. In this way, the NHI could spend less while improving diabetic peritoneal dialysis patient care by reducing the cardiovascular risk. With respect to policy decisions in the United States, it is possible that more patients would have an Hb level below 10 g/dL (ie, Hct 30%) and thus a higher cardiovascular risk might be incurred for ESRD patients with diabetes after eliminating the QIP requirement of an Hb level <10 g/dL. Thus, determining whether a lower bound of the Hct/Hg level should be restored for ESRD patients with diabetes mellitus to reach a balance between cost reduction and improvement of patient care is a critical issue to be examined by US policymakers.

### Limitations

A more clinically oriented inquiry may explain why the peritoneal dialysis patients with diabetes showed a stronger response to the increase in erythropoietin dosage and Hct levels in terms of reducing cardiovascular risk. Our data do not enable directly testing this clinical issue and thus more research to this end is warranted. There are also limitations of this study. No blood pressure or laboratory data, including serum albumin and lipid profile, were available from the NHI claim database, which prevented performing a comprehensive comparison of baseline characteristics between the two cohorts. Although this might have constrained detailed matching of patients in the two cohorts, the patients matched in the two cohorts were considerably comparable with respect to comorbid conditions and concomitant medication related to cardiovascular risk.

### Conclusions

After loosening the erythropoietin payment criteria, a significantly lower risk of cardiovascular events, stroke, and heart failure hospitalization was observed in matched cohort 2, in particular for those with diabetes mellitus. This risk reduction may be related to the higher erythropoietin dosage and maintenance of an adequate Hct range. Further research is needed to investigate why peritoneal dialysis patients with diabetes mellitus are more sensitive to the increase in erythropoietin dosage and Hct levels. Our findings support that for these patients, maintaining an Hct level above 30% is crucial for reducing the cardiovascular risk. This finding has implications for policymakers to determine the allocation of health care resources in a cost-effective manner while reducing the potential cardiovascular risk for patients receiving peritoneal dialysis.

## Abbreviations

CKD: chronic kidney disease
CMS: US Centre for Medicare and Medicaid
ESRD: end-stage renal disease
Hb: hemoglobin
Hct: hematocrit
HR: hazard ratio
ICD-9: International Classification of Diseases, Ninth Revision
NHI: Taiwan National Health Insurance
QIP: quality incentive program
PS: propensity score
